# Electrical stimulation to accelerate wound healing

**DOI:** 10.3402/dfa.v4i0.22081

**Published:** 2013-09-16

**Authors:** Gaurav Thakral, Javier LaFontaine, Bijan Najafi, Talal K. Talal, Paul Kim, Lawrence A. Lavery

**Affiliations:** 1 Department of Plastic Surgery, The University of Texas Southwestern Medical Center, Dallas, TX, USA; 2 Department of Surgery, University of Arizona, Tucson, AZ, USA; 3 Department of Medicine, Diabetic Foot and Wound Center, Hamad Medical Corporation, Doha, Qatar; 4 Department of Plastic Surgery, Georgetown University, Washington, DC, USA

**Keywords:** diabetic foot ulcer, electric stimulation therapy, treatment outcome, perfusion, infection

## Abstract

**Background:**

There are several applications of electrical stimulation described in medical literature to accelerate wound healing and improve cutaneous perfusion. This is a simple technique that could be incorporated as an adjunctive therapy in plastic surgery. The objective of this review was to evaluate the results of randomized clinical trials that use electrical stimulation for wound healing.

**Method:**

We identified 21 randomized clinical trials that used electrical stimulation for wound healing. We did not include five studies with treatment groups with less than eight subjects.

**Results:**

Electrical stimulation was associated with faster wound area reduction or a higher proportion of wounds that healed in 14 out of 16 wound randomized clinical trials. The type of electrical stimulation, waveform, and duration of therapy vary in the literature.

**Conclusion:**

Electrical stimulation has been shown to accelerate wound healing and increase cutaneous perfusion in human studies. Electrical stimulation is an adjunctive therapy that is underutilized in plastic surgery and could improve flap and graft survival, accelerate postoperative recovery, and decrease necrosis following foot reconstruction.

Electrical stimulation may offer a unique treatment option to heal complicated and recalcitrant wounds, improve flap and graft survival, and even improve surgery results. Electrical stimulation has been suggested to reduce infection, improve cellular immunity, increase perfusion, and accelerate wound healing (1).

Similar to many medical devices, electrical stimulation has a history of genuine medical application as well as quackery. In ancient Greece and Rome, ‘electric eels’ were used in footbaths to treat pain and improve blood circulation (2). In the 17th century, gold leaf was used to prevent scarring from small pox (3). Later on, gold leaves were applied directly to wounds to improve wound healing (4, 5). John Wesley, an 18th-century electrotherapist, listed cases of pain relief following electrical stimulation for suspected cases of angina, headaches, and pains in the feet (6). Currently, there is a substantial body of work that supports the effectiveness of electrical stimulation for wound healing. Treatment is safe, effective, and well tolerated. However, most surgeons have never used this approach and have a poor understanding of the technology and its potential applications. The goal of this review was to examine the results of randomized clinical trials that use electrical stimulation to accelerate wound healing.

## Types of electrical stimulation

Electrical stimulation is used for a variety of clinical applications, such as fracture repair, pain management, and wound healing. Several different applications of electricity have been described, including direct current (DC), alternating current (AC), high-voltage pulsed current (HVPC), and low-intensity direct current (LIDC). Physicians are probably most familiar with pulsed electromagnetic field (PEMF) for repair of fracture non-unions and transcutaneous electrical nerve stimulation (TENS) for pain control (7, 8). Frequency rhythmic electrical modulation systems (FREMS) is a form of transcutaneous electrotherapy using electrical stimulation that automatically varies in terms of pulse, frequency, duration, and voltage (9). Even through the electrical stimulation and wound healing literature uses several different types of electrical stimulation, they all seem to have positive results.

## Electricity and cellular proliferation

The human cell is an electrical unit. The initial measurements of the transcutaneous voltage across the human skin by Baker et al. (10) were later validated through a larger study of 17 health volunteers (11). More recent investigation has shown that all living cells are enveloped by a plasma membrane that operates on the electrochemical physiology principle of DC exchange of ions (12). Injury to the epithelial layer disrupts the body's naturally occurring electrical current therefore creating an electrical field. This electrical field, along with chemotaxis and injury stimulation, guides epithelial cell migration during wound healing (13–16). A laboratory study has shown enhanced movement of epithelial cells through application of electrical fields (*p*=0.027) (17). Movement of epithelial cells does not occur in a linear fashion; rather the cells migrate approximately along the electrical field. Cells demonstrated the ability to change direction as much by as 180° in response to electrical fields. Interesting, once cellular migration was observed, the authors reversed the polarity of the electrical field and noticed a reversal of epithelial and fibroblast migration (18). Cells cultured without exposure to an electrical field exhibited a random orientation of the long axis of cell bodies or a cobblestone morphology (19). Epithelial cells cultured in the presence of an electrical field demonstrate an increase in the distance of cell movement (*p*=0.046) (17). Under DC, endothelial cell orientation was seen as early as 4 hours after the onset of an electrical field. Longer electrical field stimulation, up to 3 days with 100 millivolts per millimeter (mV/mm), accelerated the orientation and elongation of endothelial cells compared to the control (19).

Electrical stimulation is believed to restart or accelerate wound healing by imitating the natural electrical current that occurs in injured skin. PEMF stimulation decreases the doubling time of fibroblasts and endothelial cells in culture (20). PEMF increases p42/44 mitogen-activated protein (MAP) kinase activation, which is central to initiating cell responses and leads to cell proliferation (21). Electrical stimulation applied to injured tissue increases the migration of neutrophils and macrophages and stimulates fibroblasts.

## Electricity and infection

Bacterial load and infection are thought to be important factors in chronic wounds and delayed healing (22–24). Bacterial colonization of >10^5^ organisms per gram of tissue is associated with infection and delayed wound healing in chronic wounds (25, 26). In a study by Xu et al., the rate of healing had a strong inverse relationship with log colony-forming units (CFUs). For every log order of CFUs, there was a 44% delay in wound healing (27). Halbert and Rohr took bacterial cultures from 83 limbs and showed an association between delayed wound healing and higher bacterial counts in leg ulcers. Compared to non-colonized ulcers, colonized ulcers had longer duration at presentation, larger size at presentation, and took longer time to heal (*p*<0.01) (23, 28). Electrical stimulation has the potential to be an effective adjunctive therapy to reduce bacterial load and clinical infections. Kincaid et al. showed bacteriostatic effects of HVPC *in vivo* after 2 hours at 250 V or greater on *Staphylococcus aureus, Escherichia coli*, and *Pseudomonas aeruginosa* (29). Rowley and colleagues showed a bacteriostatic effect in 81 rabbit wounds infected with *P. aeruginosa* that received low-intensity DC with a current from 0.2 to 2 µA (30). Various types of electrical stimulation have been reported to produce inhibitory effects on the growth of multiple bacterial organisms (1, 29–34). The bacteriostatic and bactericidal effects of electrical stimulation may lower the bioburden in the wound bed, therefore providing one mechanism to facilitate wound closure. Unfortunately, there are no clinical studies that report infection or adverse events in the electrical stimulation randomized controlled trials (RCTs) that we evaluated.

## Electricity and perfusion

Six randomized clinical trials were identified that evaluate the effect of electrical stimulation on perfusion (Table 1). A variety of tools were used to measure cutaneous perfusion including laser Doppler flowometry, microvessel density, and measures of transcutaneous oxygen. Five studies reported a significant increase in at least one of the measurement devices in all or a subgroup of study subjects. Clover noted a significant increase in capillary density in patients with peripheral artery disease after 3 and 6 weeks of TENS treatment and 4 weeks post-treatment (*p*<0.005). Microvessel density was determined by microscope visualization of nailfold capillaries. Perfusion was also determined by transcutaneous oxygen tension and provided measurement of skin oxygen supply in superficial vessels. Transcutaneous oxygen measurements were significantly greater in the treatment group at 3 and 6 weeks of treatment and 4 weeks post-treatment (*p*<0.05) (35). Cramp reported increased laser Doppler blood flow in a double-blinded study of healthy subjects with the application of TENS. There was a significant increase in blood flow in the low-intensity TENS group compared to the control and high-frequency TENS groups at 3, 6, 9, 12, and 15 min (*p*<0.05) after the start of treatment (36). Gilcreast evaluated perfusion in 132 diabetic subjects that were non-tobacco users, before and after electrical stimulation. A subgroup of subjects demonstrated a significant increase in transcutaneous oxygen measurement (27%, *n*=35). Responders were older, more likely to have neuropathy, higher blood glucose levels (glycated hemoglobin >9%) and good perfusion to the forefoot (toe blood pressure >70 mm Hg).

**Table 1:** Perfusion randomized controlled trial (RCT) organized by the type of ulcer

Author	Pathology of interest	Duration of treatment	Treatment specification: voltage, current, phase duration, frequency	Population	Outcome
Gilcreast (45)	Perfusion in DFU and high-risk population using HPVC	Once Span: 1 day	100 V, 100 pps, 0.07 pulse duration	Treatment *n*=132	TcpO2 significant improvement in 27% of subjects (*p*<0.05). No change in 73% of study subjects. Laser Doppler flow NS. Capillary density NS.
Clover (35)	Perfusion in stable claudication using TENS	1 hour, TID, for 6 weeks Span: 6 weeks	1.0 V, 10 mA, 8 Hz	Treatment *n*=24, Control: *n*=12	Capillary density increased treatment 25% vs. control 0% *p*<0.005 TcpO2 was greater in treatment group vs. control, *p*>0.05, raw value NS. Laser Doppler flow NS.
Cramp (36)	Perfusion in health humans using TENS	Once, 15 min Span: 1 day	High frequency = 110 Hz, 200 µs Low frequency 4 Hz, 200 µs	High frequency *n*=10 Low frequency *n*=10 Sham** *n*=10	TcpO2 NS. Laser Doppler blood flow was greater in the low-frequency group compared vs. other groups *p*=0.01. Capillary density NS.
Forst (46)	Perfusion in neuropathic patients using TENS	Once, 3 min Span: 1 day	0.2 ms at 4 cycles/s 70 mA or painless muscle contraction	NP-/RP− *n*=14, NP + /RP− *n*=14, NP − /RP+ *n*=8, NP + /RP + *n*=21, Non-diabetic *n*=21	TcpO2 NS. Laser Doppler blood flow increased with ES in all groups at the dorsum of the foot *p*>0.05. Capillary density NS.
Peters (44)	Perfusion in diabetics using DC	60 min, QID, for 1 day Span: 2 days	50 V, 100 twin-peak monophasic pps	Diabetics with PAD *n*= 11 and without PAD *n*=8	TcpO2 significant improvement in patients with PAD 27% (*p*<0.05) No change in patients without PAD. Laser Doppler blood flow no difference (*p*=0.27) Capillary density NS.
Griffin (41)	Venous flow with TENS	Twelve increments in stimuli per minute (spm)	0–5 V, 50 ms, 2–120 spm	Healthy volunteers *n =* 24	Peak systolic velocity in popliteal artery was 10 times higher at 2–8 spm than baseline Ejection volume was 19 times higher at spm than 120 spm.

* Single-blind RCT;

** double-blind RCT; NS, not stated; pps: pulse per second.

In addition to increased skin perfusion, electrical stimulation therapy has been shown to improve venous flow (37–40). TENS was evaluated in 24 healthy individuals and was shown to increase the activity of the calf muscle pump. At baseline, the mean popliteal vein peak systolic velocity was 10 cm/s. From 2 to 8 stimuli per minute (spm), the peak systolic velocity increased to 96–105 cm/s, roughly 10 times higher. As the spm was increased to 120 cm/s, there was a decrease in peak systolic velocity to 35 cm/s. A similar occurrence was observed with ejection volume. There was a 19-fold increase in the ejection volume of the popliteal vein at two pulse per second (pps) compared to 120 pps. However, as spm increased, the ejected volume per minute increased 12 times from 20 to 240 ml/min (41). The benefits of TENS in supplementing calf muscle pump may be dose-dependent. In the standing or upright position, the higher peak systolic velocity from a low stimuli frequency would benefit patients to overcome backflow secondary to gravity. During leg elevation, gravity is partially compensated, therefore a high stimuli frequency would allow for rapid edema reduction through an increased ejected volume per minute. It is possible to benefit from electrical stimulation during the inactive and active course of the day.

Increased perfusion associated with electrical stimulation may be associated with increased vascular endothelial growth factor (VEGF). Kanno and colleagues evaluated the effect of electrical stimulation on (VEGF). VEGF is a growth factor thought to be a primary angiogenic factor. The expression of VEGF is unregulated by hypoxia and cytokines. Kanno et al. used cultured skeletal muscle cells that were exposed to non-contractile pulsed electrical stimulation for 24 hours. Cells were exposed to 2 hours of electrical stimulation and VEGF mRNA expression was measured at 24 hours. Almost identical mRNA expression was seen between the transient and continuous electrical stimulation after 24 hours. VEGF mRNA returned to basal levels 46 hours after 2 hours of treatment with electrical stimulation (42). Zhao et al. applied an electrical field of 200 mV/mm, the same as the measured skin wounds, to cells in a culture and noted a significant increase in VEGF released into the culture medium. This elevation in VEGF occurred as early as 5 min after exposure to an electrical field followed by a reduction in levels at 1 and 2 hours. Levels of VEGF rose again at 4 hours. After 24 hours of electrical stimulation, VEGF levels were at their highest (19).

The full benefits of electrical stimulation on perfusion may not be realized after a single treatment, since gene expression reverts to basal levels after a short duration. Electrical stimulation may have a bimodal effect on perfusion through an initial release of stored VEGF followed by a later increase in gene expression of VEGF. Daily electrical stimulation might allow secretion of VEGF to remain above basal levels throughout the healing process. However, several studies reported a significant improvement in cutaneous perfusion very quickly (36, 43–46). For instance, Peters noted that subjects with peripheral arterial disease (PAD) had a significant increase in perfusion within the first 5 min of therapy (*p*=0.040); however, patients without PAD did not have a change in cutaneous blood flow (44).

## Electrical stimulation and wound healing

We initially identified 21 RCTs that used electrical stimulation to treat wounds. A literature review was planned and performed in Medline. The following search strategy was used in the PubMed database: ‘electrical stimulation’ [Mesh] and ‘wound healing’ [Mesh]. Titles and abstracts were screened and full texts were analyzed for meeting the inclusion criteria. Only randomized clinical trials in humans were included. Case studies and clinical trials focused on children and the congenital disability were excluded. Out of these studies, five were excluded because they had less than eight subjects in the treatment groups (47–51). We evaluated 16 randomized clinical studies that used a variety of different applications of electrical stimulation to treat wounds (Table 2) (7, 8, 52, 53). Electrical stimulation has been evaluated in pressure ulcers, venous stasis ulcers, vascular ulcers, and diabetic foot wounds (Table 2). One of the challenges in interpreting these data is the variation in outcome measurements, type of electrical stimulation, and how therapy was dosed in the trials. Most of the studies were small and probably underpowered; many studies had a short treatment period (less than 8 weeks, *n*=11, mean 3.1 weeks, 8–12 weeks long, *n*=3, or 12 or more weeks: *n*=6, mean 12.6 weeks) (Table 2). In addition, many of the studies did not use complete wound healing as the primary outcome. Because of the short duration of the studies, change in wound area was often used instead of wound healing (Table 2). Out of 16 wound healing studies, 8 studies reported both wound healing and wound area reduction.

**Table 2:** Wound healing RCT organized by the type of ulcer

Author	Pathology of interest	Duration of treatment	Treatment specification; voltage, current, phase duration, frequency	Population	Outcome
Peters (54)	DFU using DC	8 hours, nightly, for 12 weeks Span: 12 weeks	50 V, 80 twin-peak monophasic pps for 10 min, 8 pps for 10 min, then 40 min standby cycles	Treatment *n*=20. Sham** *n*=20	Wound healing ES 65% vs. sham 35% *p*=0.058. Wound area reduction ES 86% vs. sham 71% *p*>0.05. Adverse Event: 10% ES and 15% sham infection.
Adunsky (56)	Pressure ulcers using DC	20 min, TID, 7 day a week, for 2 weeks. Then BID for 6 weeks Span: 8 weeks	NS	Treatment *n*=19 Sham** *n*=19	Wound healing ES 26% vs. sham 16% *p*=0.39. Wound area reduction ES 31% vs. sham 4% *p*=0.9. Adverse events: 14% ES and 18% sham medical reasons. 31% ES and 14% sham had clinical deterioration, consent withdrawal or technical difficulties.
Griffin (57)	Pressure ulcers ion males using HVPC	60 min, daily, for 20 consecutive days Span: 20 days	200 V, total current 500 µA, 100 pps	Treatment *n*=8 Sham* *n*=9	Wound healing ES 38% s 22% *p*>0.05. Wound area reduction was greater in ES group vs. sham *p*=.05, raw value NS. Adverse events: NS.
Houghton (58)	Pressure ulcers using HVPC	60 min, TID, for 3 months. Span: 3 months	50–150 V. 50 µs pulses. 20-min intervals at 100 Hz, 10 Hz, then off cycle Polarity was alternated weekly	Treatment *n*=16, Sham* *n*=18	Wound healing ES 38% vs. control 28% *p*>0.05. Wound area reduction ES 70% vs. control 36% *p*=.048. Adverse events: NS.
Salzberg (59)	Pressure ulcers in males using PEMF	30 min, BID, 7 days a week, for 12 weeks Span: 12 weeks	Radio frequency of 27.12 MHz, 80–600 pps, a duty cycle between 0.5–3.9% and 293–975 W	Treatment *n*=9. Sham** *n*=10	Wound healing ES 100%, average 14 days vs. sham 100%, average 35 days *p*=0.007. Wound area reduction NS. Adverse events: 10% ES patients were missing data.
Wood (60)	Pressure ulcer using DC.	Three time a week, for 8 weeks. Span: 8 weeks	600 µA, 0.8 Hz.	Treatment *n*=41 Shams** *n*=30	Wound healing ES 58% vs. sham 3% *p*<0.0001. Wound area reduction NS. Adverse events: NS.
Ieran (61)	Venous ulcers using PEMF	3–4 hours, daily, 7 days a week, for 90 days. Span: 90 days	2.8 mT, 75 Hz, 1.3-ms pulse width	Treatment *n =* 18 Sham** *n =* 19	Wound healing ES 67% vs. sham 32% *p*<0.02. Wound area reduction ES 47% vs. sham 30%, *p*>0.05. Adverse event: 9% ES and 14% sham non-compliance, 5% ES allergic reaction, and 5% ES was diagnosed with rheumatoid arthritis.
Lundeberg (62)	Venous ulcers using AC	20 min, BID, for 12 weeks. Span: 12 weeks	80 Hz, 1-ms pulse width. Polarity was reversed after each treatment	Treatment *n*=24 Sham* *n*=27	Wound healing ES 41% vs. sham 15% *p*<0.05. Wound area reduction ES 59% vs. sham 39% *p*<0.05. Adverse event: 6% ES and 3% sham had allergy, 9% ES and 6% sham had pain, 9% ES and 6% sham non-compliant.
Stiller (20)	Venous ulcers using PEMF	3 hours, daily, 7 days a week, for 8 weeks. Span: 8 weeks	0.06 mV/cm. The signal is 3-part pulse (+, −, +) of 3.5-ms width	Treatment *n*=18, Sham** *n*=13	Wound healing NS. Wound area reduction ES 48% vs. control 42% increase *p*<0.0002. Adverse event: No events.
Santamato (9)	Venous leg ulcer healing using FREMS	25 min, 5 days a week, 3 weeks Span: 3 weeks	Maximum impulse amplitude preset to the value according to patient's sensitivity threshold	Treatment *n*=10 Control *n*=10	Wound healing NS. Wound area reduction ES (58%) vs. control (25%) (*p*<0.005). Adverse events: none.
Carley (8)	Mixed ulcers using DC	2 hours, BID, 5 days a week, for 5 weeks. Span: 5 weeks	300–500 µA for normally innervated and 500–700 µA for denervated skin 30–110 µA/cm^2^	Treatment *n*=15, Control *n*=15.	Wound healing NS. Wound area reduction ES 89% vs. control 37% *p*<0.01. Adverse event: NS.
Feedar (53)	Mixed ulcer using pulsed DC	30 min, BID, 7 days a week, for 4 weeks. Span: 4 weeks	29.2 V, maximum 29.2 µA, 128 pps. Polarity reversed every 3 days until stage II was reached, then daily reversal with 64 pps	Treatment *n*=26 Sham** *n*=24	Wound healing ES 0% vs. sham 4%, *p*>0.05. Wound area reduction ES 66% vs. shams 33% *p*<0.02. Adverse event: NS.
Houghton (63)	Mixed ulcers using HVPC	45 min, 3 times a week, for 4 weeks. Span: 4 weeks	150 V, 100 µs, 100 Hz	Treatment *n*=14 Sham** *n*=13	Wound healing NS. Wound area reduction ES 44% vs. sham 16% *p*<0.05. Adverse event: NS.
Jankovic (64)	Mixed ulcers using FREMS	40 min, daily, 5 days a week, for 3 weeks Span: 3 weeks	300 V, 1,000 Hz, 10–40 µs, 100–170µA.	Treatment *n*=20 Control *n*=15	Wound healing NS. Wound area reduction ES 82% vs. control 46% *p*<0.001. Adverse event: NS.
Lawson (65)	Mixed wounds using DC	30 min, three times a week, for 4 weeks. Span: 4 weeks	5 V, 30 Hz, pulse width 200 µs. Current of 20 mA	DM I or II: *n*=8 Without DM: *n*=9	Wound healing NS. Wound area reduction diabetics 70% non-diabetics 38% *p*<0.01. Adverse event: 20% of diabetic group was hospitalized. Ten percent of non-diabetic dropped out secondary to vertigo.
Sarma (55)	Leprosy ulcers using PEMF	30 min, daily, 5 days a week, for 35 days. Span: 35 days	Sinusoidal form 0.95–1.05 Hz; amplitude ± 2,400 nT	Treatment *n*=18 Sham** *n*=15	Wound healing ES 6% vs. sham 0%, *p*>0.05. Wound volume reduction ES 86% vs. sham 48% *p*=0.04. Adverse event: 10% ES and 10% sham removed for irregularity in attendance and 15% sham removed for suspicion of malignancy.

* Single-blind RCT

** double-blind RCT; NS, not stated; pps, pulse per second; NP, neuropathy; RP, retinopathy.

A few investigators suggested that compliance may be a factor that affects wound healing in electrical stimulation studies (54, 55) (Table 2). However, in most electrical stimulation studies, therapy was provided in a hospital or clinic setting, so patients keeping their clinic appointment determined the main measure of compliance. The study by Peters et al. was the only study that provided an electrical stimulation device for study patients to use at home. Peters et al. recorded the number of hours the electrical stimulation device was used. These data were downloaded from the electrical stimulation device at weekly clinic visits. There was no significant difference in the compliance rates between the two treatment groups. Peters further stratified the results based on compliance. There was a trend demonstrating a dose response with electrical stimulation. A higher proportion of wounds healed in compliant patients in the electrical stimulation treatment group (71%), non-compliant patients in the electrical stimulation treatment group (50%), compliant patients in the sham group (39%), and non-compliant patients in the sham group (29%) (54). Sarma et al. excluded patients from their analysis due to irregularities in attendance rather than including all subjects in an intent-to-treat analysis (55). Non-compliance is a universal concern in clinical practice. Most electrical stimulation devices do not provide any mechanism to evaluate the duration that the therapy was actually used by the patient. However, it would certainly be advantageous for physicians to have this information to educate the patient and document treatment compliance.

There are two inconclusive studies with electrical stimulation and wound healing. Both of these studies had a small sample size (40 and 38 subjects) and were underpowered (54, 56). First, Peters studied 40 patients with diabetic foot ulcers for 12 weeks. Patients were randomized to receive HVPC or sham therapy. This study had the most frequent dosing of electrical stimulation. Patients received 20 min of electrical stimulation every hour for 8 hours each day over the 12-week study. More patients healed in the electrical stimulation group (65% compared to the sham group 35%), but the difference was not significant (*p*=0.058). However, when patient compliance was evaluated, patients that used the device at least three times a week were more likely to heal than patients that received sham therapy and patients who used electrical stimulation 0, 1, or 2 times a week (*p*=0.038) (54). Adunsky reported the second study. Thirty-eight patients with pressure ulcers were distributed equally between shams and treatment with DC application of electrical stimulation for 8 weeks. The primary outcome was percent change in wound area, and despite the small sample size, the results were almost significant (wound area reduction 31% vs. 4%, *p*=0.09) (56).

## Study limitations

There are several limitations to this review. There were several different applications of electrical stimulation (PEMF, TENS, high voltage galvanic stimulation), different doses, and durations of therapy that were studied and reported. In addition, many of the studies were small and may have been underpowered. And unlike industry-sponsored phase-three clinical trials, many studies looked at percent change in wound area at 4–6 weeks as the primary outcome rather than complete wound healing at 12 or 20 weeks. Despite variations in the type of current, duration, and dosing of electrical stimulation, the majority of trials showed a significant improvement in wound area reduction or wound healing compared to the standard of care or sham therapy (Table 2) as well as improved local perfusion (Table 1). In fact, these factors were different in all 16 RCTs.

## Conclusion

There are many opportunities to improve clinical outcomes with electrical stimulation. In many ways, electrical stimulation appears to be a perfect adjunctive therapy. First, no device-related complications or adverse effects have been reported in the existing literature. The therapy is safe and easy to use. Second, as electrical stimulation decreases bacterial infection, increases local perfusion, and accelerates wound healing, it addresses these three pivotal factors in surgical wound complications. Electrical stimulation offers a unique treatment option to heal complicated and recalcitrant wounds, improve flap, replantation and graft survival, and even improve surgery results. This is an approach that can be applied in the operating room and used throughout the recovery process. Electrical stimulation is a simple, inexpensive intervention to improve surgical wound healing. Rigorous clinical trials are needed to help understand the dosing, timing, and type of electrical stimulation to be used.
